# Supplementary material for: Resolving the full spectrum of human genome variation using Linked-Reads
Source: Genome Res. 2019 Apr;29(4):635–45. doi: 10.1101/gr.234443.118 (PMC6442396; doi:10.1101/gr.234443.118)
Supplement: Supplemental Material [file supp_gr.234443.118_Supplemental_longranger-2.2.zip › longranger-2.2.tar.bz2!/lr2.2-public/lib/stan/cmdstan/stan/doc/api/html/stepsize__adaptation_8hpp_source.html]

Stan: src/stan/mcmc/stepsize\_adaptation.hpp Source File


|  |  |
| --- | --- |
|  | Stan  2.5.0  probability, sampling & optimization |


- Main Page
- Related Pages
- Namespaces
- Classes
- Files

- File List
- File Members

All Classes Namespaces Files Functions Variables Typedefs Enumerations Enumerator Friends Macros Pages

- src
- stan
- mcmc

stepsize\_adaptation.hpp

Go to the documentation of this file.

1 #ifndef STAN\_\_MCMC\_\_STEPSIZE\_\_ADAPTATION\_\_BETA

2 #define STAN\_\_MCMC\_\_STEPSIZE\_\_ADAPTATION\_\_BETA

3

4 #include <cmath>

5 #include <stan/mcmc/base\_adaptation.hpp>

6

7 namespace stan {

8

9  namespace mcmc {

10

11  class stepsize\_adaptation: public base\_adaptation {

12

13  public:

14

15  stepsize\_adaptation(): mu\_(0.5), delta\_(0.5), gamma\_(0.05),

16  kappa\_(0.75), t0\_(10)

17  { restart(); }

18

19  void set\_mu(double m) { mu\_ = m; }

20  void set\_delta(double d) { if(d > 0 && d < 1) delta\_ = d; }

21  void set\_gamma(double g) { if(g > 0) gamma\_ = g; }

22  void set\_kappa(double k) { if(k > 0) kappa\_ = k; }

23  void set\_t0(double t) { if(t > 0) t0\_ = t; }

24

25  double get\_mu() { return mu\_; }

26  double get\_delta() { return delta\_; }

27  double get\_gamma() { return gamma\_; }

28  double get\_kappa() { return kappa\_; }

29  double get\_t0() { return t0\_; }

30

31  void restart() {

32  counter\_ = 0;

33  s\_bar\_ = 0;

34  x\_bar\_ = 0;

35  }

36

37  void learn\_stepsize(double& epsilon, double adapt\_stat) {

38

39  ++counter\_;

40

41  adapt\_stat = adapt\_stat > 1 ? 1 : adapt\_stat;

42

43  // Nesterov Dual-Averaging of log(epsilon)

44  const double eta = 1.0 / (counter\_ + t0\_);

45

46  s\_bar\_ = (1.0 - eta) \* s\_bar\_ + eta \* (delta\_ - adapt\_stat);

47

48  const double x = mu\_ - s\_bar\_ \* std::sqrt(counter\_) / gamma\_;

49  const double x\_eta = std::pow(counter\_, - kappa\_);

50

51  x\_bar\_ = (1.0 - x\_eta) \* x\_bar\_ + x\_eta \* x;

52

53  epsilon = std::exp(x);

54

55  }

56

57  void complete\_adaptation(double& epsilon) {

58  epsilon = std::exp(x\_bar\_);

59  }

60

61  protected:

62

63  double counter\_; // Adaptation iteration

64  double s\_bar\_; // Moving average statistic

65  double x\_bar\_; // Moving average parameter

66  double mu\_; // Asymptotic mean of parameter

67  double delta\_; // Target value of statistic

68  double gamma\_; // Adaptation scaling

69  double kappa\_; // Adaptation shrinkage

70  double t0\_; // Effective starting iteration

71

72  };

73

74  } // mcmc

75

76 } // stan

77

78 #endif

stan::mcmc::stepsize\_adaptation::complete\_adaptation

void complete\_adaptation(double &epsilon)

**Definition:** stepsize\_adaptation.hpp:57

stan::mcmc::stepsize\_adaptation::delta\_

double delta\_

**Definition:** stepsize\_adaptation.hpp:67

stan::mcmc::stepsize\_adaptation::restart

void restart()

**Definition:** stepsize\_adaptation.hpp:31

base\_adaptation.hpp

stan::mcmc::stepsize\_adaptation

**Definition:** stepsize\_adaptation.hpp:11

stan::mcmc::stepsize\_adaptation::counter\_

double counter\_

**Definition:** stepsize\_adaptation.hpp:63

stan::mcmc::stepsize\_adaptation::set\_t0

void set\_t0(double t)

**Definition:** stepsize\_adaptation.hpp:23

stan::agrad::pow

fvar< T > pow(const fvar< T > &x1, const fvar< T > &x2)

**Definition:** pow.hpp:17

stan::mcmc::stepsize\_adaptation::learn\_stepsize

void learn\_stepsize(double &epsilon, double adapt\_stat)

**Definition:** stepsize\_adaptation.hpp:37

stan::mcmc::stepsize\_adaptation::kappa\_

double kappa\_

**Definition:** stepsize\_adaptation.hpp:69

stan::mcmc::stepsize\_adaptation::set\_mu

void set\_mu(double m)

**Definition:** stepsize\_adaptation.hpp:19

stan::mcmc::stepsize\_adaptation::get\_mu

double get\_mu()

**Definition:** stepsize\_adaptation.hpp:25

stan::mcmc::stepsize\_adaptation::mu\_

double mu\_

**Definition:** stepsize\_adaptation.hpp:66

stan::mcmc::stepsize\_adaptation::get\_kappa

double get\_kappa()

**Definition:** stepsize\_adaptation.hpp:28

stan::mcmc::stepsize\_adaptation::x\_bar\_

double x\_bar\_

**Definition:** stepsize\_adaptation.hpp:65

stan::agrad::sqrt

fvar< T > sqrt(const fvar< T > &x)

**Definition:** sqrt.hpp:15

stan::mcmc::stepsize\_adaptation::stepsize\_adaptation

stepsize\_adaptation()

**Definition:** stepsize\_adaptation.hpp:15

stan::mcmc::stepsize\_adaptation::s\_bar\_

double s\_bar\_

**Definition:** stepsize\_adaptation.hpp:64

stan::mcmc::stepsize\_adaptation::gamma\_

double gamma\_

**Definition:** stepsize\_adaptation.hpp:68

stan::mcmc::stepsize\_adaptation::set\_gamma

void set\_gamma(double g)

**Definition:** stepsize\_adaptation.hpp:21

stan::mcmc::stepsize\_adaptation::set\_kappa

void set\_kappa(double k)

**Definition:** stepsize\_adaptation.hpp:22

stan::mcmc::base\_adaptation

**Definition:** base\_adaptation.hpp:8

stan::mcmc::stepsize\_adaptation::get\_t0

double get\_t0()

**Definition:** stepsize\_adaptation.hpp:29

stan::mcmc::stepsize\_adaptation::get\_gamma

double get\_gamma()

**Definition:** stepsize\_adaptation.hpp:27

stan::mcmc::stepsize\_adaptation::get\_delta

double get\_delta()

**Definition:** stepsize\_adaptation.hpp:26

stan::agrad::exp

fvar< T > exp(const fvar< T > &x)

**Definition:** exp.hpp:16

stan::mcmc::stepsize\_adaptation::t0\_

double t0\_

**Definition:** stepsize\_adaptation.hpp:70

stan::mcmc::stepsize\_adaptation::set\_delta

void set\_delta(double d)

**Definition:** stepsize\_adaptation.hpp:20


---

[ Stan Home Page ]

*© 2011–2014,
Stan Development Team.*
